# Case of Perinephritic Abscess

**Published:** 1887-04

**Authors:** Harlow N. Orton

**Affiliations:** Minneapolis, Minn.


### DOMESTIC CORRESPONDENCE.

PERINEPHRITIC ABSCESS, *with absence of constitutional symptoms of suppuration.* HARLOW N. ORTON, M.D.

When pus in large quantities is being formed, or exudated, we generally expect the patient to exhibit some constitutional disturbance, as rigors, elevation, temperature, etc.; but once in a while there comes under our observation an exception to this rule: a human system peculiarly prone to suppuration: In pus-formers, an anomaly like the "bleeder-families" (Hæmophilia), no cause is known, except hereditary predisposition. It is in this class of cases we are apt to find the symptoms denoting suppuration somewhat obscured. In illustration, I will relate the history of a case.

Jacob Mosden, age 24, married; tall, muscular man; occupation, switchman on the N. P. R. R. Said he had been pretty healthy since ten years of age; before that time had been troubled with large abscesses on different parts of his body.

On September 1st., 1886, he sprained his wrist, and in two or three days had cellular inflammation of the whole forearm. He was treated in the hospital at Brainerd, Minnesota, for several weeks. Five openings were made, and a large amount of pus drained off.

He called at my office on October 2nd. His arm was about well, but his general health was not very good. I prescribed tonics, and he improved rapidly. On October

15th I was called to his house, and found him down on his back again. Examination revealed acute pneumonia with consolidation of lower lobe of the right lung, from which he soon recovered, and on November 12th he was feeling well, and was about to start to work.

On November 13th I was called to his house again, and he complained of pain a little above the region of the right kidney. He had no rigors, and I found no rise of temperature. There was slight prominence of the muscles, and some tenderness a little above the region of the kidney. His urine proved to be normal on examination. My diagnosis was neuralgia, for the want of something better to call it. I prescribed a mercurial purgative; quinine, 5 grains three times a day; and an anodyne liniment for his back.

On November 16th the parts were very much swollen and reddened. He had no chills or fever. I concluded that a perinephritic abscess was being formed, and advised the continuous application of hot poultices over the swelling.

On November 19th his condition was about the same. No fluctuation could be detected thus far. Dr. J. W. Bell was called in consultation, and concurred in my diagnosis.

On November 23rd his temperature was 101, and his pulse 85. He had no chills at any time. His urine was normal, the swelling increased to about twice the size it was at the last examination, fluctuation was just perceptible. I cut down upon the abscess at the outer border of the quadratus lumborum muscle, and made a free opening of about one inch and a half. This let out over a quart of pus; a simple poultice being applied over the surface. In the next twelve hours it discharged about one pint more; no drainage tube having been used, the incision was free enough to admit of a ready outlet for the contents, and there was no re-accumulation.

I think it was due to his peculiar diathesis that he did not exhibit some general symptoms of systemic poisoning, which that amount of pus in any part of the body generally produces.

Minneapolis, Minn.

